# Regional and rural-urban patterns in the prevalence of diagnosed hypertension among older U.S. adults with diabetes, 2005–2017

**DOI:** 10.1186/s12889-024-18802-5

**Published:** 2024-05-16

**Authors:** Jalal Uddin, Sha Zhu, Gargya Malla, Emily B. Levitan, Deborah B. Rolka, April P. Carson, D. Leann Long

**Affiliations:** 1 Department of Community Health and Epidemiology, Dalhousie University, 5790 University Ave, Halifax, Canada; 2 Department of Epidemiology, University of Alabama at Birmingham, Birmingham, USA; 3 Division of Diabetes Translation, National Center for Chronic Disease Prevention and Health Promotion, Centers for Disease Control and Prevention, Georgia, USA; 4 Department of Medicine, University of Mississippi Medical Center, Jackson, USA; 5 Department of Biostatistics and Data Science, Wake Forest University, Winston-Salem, USA

**Keywords:** Hypertension, Rural-urban classification, Region, Medicare, Diabetes

## Abstract

**Background:**

Hypertension prevalence among the overall US adult population has been relatively stable during the last two decades. However, whether this stabilization has occurred across rural-urban communities and across different geographic regions is unknown, particularly among older adults with diabetes who are likely to have concomitant cardiovascular risk factors.

**Methods:**

This serial cross-sectional analysis used the 5% national sample of Medicare administrative claims data (*n* = 3,516,541) to examine temporal trends (2005–2017) in diagnosed hypertension among older adults with diabetes, across urban-rural communities and US census regions (Northeast, Midwest, South, and West). Joinpoint regression was used to obtain annual percent change (APC) in hypertension prevalence across rural-urban communities and geographic regions, and multivariable adjusted regression was used to assess associations between rural-urban communities and hypertension prevalence.

**Results:**

The APC in the prevalence of hypertension was higher during 2005–2010, and there was a slowdown in the increase during 2011–2017 across all regions, with significant variations across rural-urban communities within each of the regions. In the regression analysis, in the adjusted model, older adults living in non-core (most rural) areas in the Midwest (PR = 0.988, 95% CI: 0.981–0.995) and West (PR = 0.935, 95% CI: 0.923–0.946) had lower hypertension prevalence than their regional counterparts living in large central metro areas.

**Conclusions:**

Although the magnitudes of these associations are small, differences in hypertension prevalence across rural-urban areas and geographic regions may have implications for targeted interventions to improve chronic disease prevention and management.

**Supplementary Information:**

The online version contains supplementary material available at 10.1186/s12889-024-18802-5.

## Introduction

Rural-urban disparities in health are well-documented in the US, with persons in rural communities having an excess burden of preventable diseases and mortality [1]. National data from the US Centers for Disease Control and Prevention Wide-ranging Online Data for Epidemiologic Research (CDC WONDER) demonstrated that rural-urban disparities in age-adjusted all-cause mortality rates [2] and mortality from cardiovascular disease [3] persisted from the late 1990s to early 2010s, although national mortality rates generally declined during this time. Furthermore, over the last two decades, reductions in mortality from ischemic heart disease have been slower for adults aged 65 years living in rural areas than those in urban areas [4]. 

The prevalence of hypertension increases with age, such that 77% of US adults aged 65 years have hypertension compared to about 29% of US adults aged 20–44 years [5]. Moreover, blood pressure control among those with hypertension has declined in recent years [6], further underscoring the importance of understanding trends in the burden of hypertension.

Existing studies show a mixed pattern in rural-urban differences in cardiovascular risk factors, including hypertension and diabetes. Findings from a contemporaneous cohort study with representation from the contiguous US showed that adults in rural areas were more likely to have hypertension than those in urban areas [7]. In a separate study using data from primary care practices in the Midwest, adults with diabetes living in rural areas were less likely to have controlled blood pressure than adults with diabetes living in urban areas [8]. In contrast, in the National Health and Nutrition Examination Survey data (2013–2018), the prevalence of stage II hypertension (BP ≥ 140/90 mm Hg) was higher for adults residing in medium to small metropolitan statistical areas (MSAs) but not for those residing in non-MSAs [9] Further, CDC’s Diabetes LEAD (Location, Environmental Attributes, and Disparities) Network studies have shown that the new onset type 2 diabetes (T2D) by rural-urban community types varies by region of the US. For instance, the T2D onset was higher in rural areas in the South and in high density urban communities in the Northeast region (the Geisinger sample) [10]. As hypertension and diabetes are often concomitant [11], identifying communities that may be differentially impacted by this excess burden is needed.

It is well-documented that diabetes and hypertension, separately, are more prevalent in the southern areas of the US [12–15]. While hypertension-related cardiovascular mortality rates increased in both rural and urban areas in the US from 2007 to 2017, this increase was more pronounced in rural areas in the South [16]. However, evidence is limited on rural-urban disparities in hypertension prevalence over time and across geographic regions among older adults with diabetes, a high-risk group for cardiovascular morbidity and mortality. To address this important gap in the literature, using national level Medicare administrative claims (2005–2017) data, we examined trends in diagnosed hypertension among older adults with diabetes by rural-urban residence and geographic region.

## Methods

This serial cross-sectional analysis used the 5% national random sample of Medicare data from 2005 to 2017. Medicare is a national program that provides health care insurance to US adults aged ≥ 65 years, and adults with a disability or end-stage renal disease, regardless of age. This analysis included fee-for-service beneficiaries aged ≥ 66 years with at least 12 months of continuous coverage for inpatient (Medicare Part A) and outpatient (Medicare Part B) care, and with a diagnosis for diabetes. As we restricted our analysis to those who were on Medicare for at least 12 months, the lowest age of the participants was 66. Diabetes diagnosis was determined using International Classification of Diseases codes (ICD-9 and ICD-10) from one inpatient claim or two outpatient or carrier claims that were at least seven days apart. Hypertension diagnosis was determined using similar methods. The ICD-9 and ICD-10 codes for diabetes and hypertension, respectively, were obtained from ICD manuals (Supplementary table S1) [17, 18]. 

### Regions and rural-urban classification

Geographic regions were categorized according to US Census regions (Northeast, Midwest, South, and West). For rural and urban designations, the 2013 CDC National Center for Health Statistics (NCHS) classification scheme [19] was used to categorize communities at the county-level. The NCHS uses data from the US Census and the Office of Management and Budget to classify counties into the following community types: (1) large central metropolitan; (2) large fringe metropolitan; (3) medium metropolitan; (4) small metropolitan; (5) micropolitan; and (6) and non-core. Large central metropolitan is the most urban classification, and non-core is the most rural classification. The details of the methodology and definitions of these classifications were described previously [19]. 

### Covariates

Covariates included age (66–69, 70–74, 75–79, 80–84, and 85 years), sex (male, female), race (White, Black, and Other), and dual eligibility for Medicare and Medicaid; these were obtained from the Medicare Beneficiary Summary File. Additionally, the social vulnerability index (SVI) was also included; this index ranks the relative vulnerability of a geographic area (e.g., county) based on socioeconomic status, household composition and disability, race/ethnicity composition/language, and housing/transportation. We linked publicly available 2010 SVI data to Medicare claims data. The 2010 SVI is based on the 2010 census data and SVI scores ranged from 0 to 1, with a higher score representing higher levels of vulnerability [20, 21]. 

### Statistical analysis

We examined sociodemographic characteristics across calendar years. *P*-value for trend for categorical/ordinal variables was obtained using Mantel-Haenszel Chi-square statistics [22]. Specifically, non-zero correlation was tested for ordinal covariates (e.g., age group) and linear shift was tested for nominal covariates (e.g., race, sex). Joinpoint regression was used to estimate the annual percent change (APC) in hypertension prevalence [23–25] by rural-urban community types in each census region. Briefly, joinpoint regression assesses changes in slopes for the outcome of interest between time points across a study period. Estimates were age-standardized to the 2010 US census. Poisson regression with robust standard errors was used to obtain prevalence ratios (PR) to assess the associations of rural-urban categories with hypertension in each census region. Models were unadjusted; adjusted for age, sex, race, and Medicaid-Medicare dual eligibility; and further adjusted for the 2010 social vulnerability index. A two-tailed *P* < 0.05 was considered statistically significant. Statistical analyses were conducted using SAS version 9.4 (SAS Institute Inc, Cary, NC).

## Results

### Characteristics of medicare beneficiaries 2005–2017

Table 1 presents sociodemographic characteristics by calendar year. The percentage of study participants aged 66–69 years increased from 2005 to 2017, while the percentages for those aged 75–79 and 80–84 years decreased during this time (*P*-value for trend < 0.0001). Women comprised more than half of the study participants across all calendar years, and their percentages decreased from 56.8 in 2005 to 52.8 in 2017 (*P*-value for trend < 0.0001). The percentage of study participants in the South and West regions increased from 2005 to 2017, whereas the percentages in the Northeast and Midwest decreased. For community types, there were no significant changes during the study period (*p* = 0.86).

**Table 1:** Characteristics of Medicare Beneficiaries with Diabetes (Medicare 5% Sample, 2005–2017) (*n* = 3,516,541)

Characteristics	Calendar Year													*P* value for trend
	2005	2006	2007	2008	2009	2010	2011	2012	2013	2014	2015	2016	2017	
Total Number of Beneficiaries	250,594	254,921	257,608	257,858	263,035	271,887	278,730	277,743	279,129	278,795	282,262	282,545	281,434	
**Age [years; mean (SD)]**	75.5 (6.7)	75.6 (6.7)	75.7 (6.8)	75.7 (6.8)	75.7 (6.9)	75.7 (7.0)	75.7 (7.0)	75.8 (7.0)	75.7 (7.1)	75.6 (7.1)	75.5 (7.1)	75.4 (7.0)	75.4 (7.0)	< 0.0001
**Age group [%]**														
66–69 years	22.4	22.4	22.3	22.2	22.2	22.8	23.0	23.0	23.1	23.8	24.3	25.1	25.0	< 0.0001
70–74 years	26.4	26.0	26.0	26.1	26.5	26.3	26.2	26.3	26.6	26.6	26.7	26.7	27.4	
75–79 years	23.8	23.6	23.1	22.6	21.8	21.4	21.3	21.5	21.3	21.3	21.3	21.1	20.9	
80–84 years	16.8	17.0	17.0	17.0	17.0	16.6	16.4	16.1	15.7	15.0	14.7	14.4	14.3	
>=85 years	10.6	11.1	11.6	12.1	12.5	12.9	13.1	13.2	13.3	13.2	13.0	12.7	12.4	
**Race**														
White	82.2	82.5	82.6	82.4	82.0	81.6	81.3	81.2	81.0	81.1	80.9	80.4	80.0	< 0.0001
Black	11.7	11.4	11.0	10.9	11.1	11.2	11.4	11.3	11.3	11.1	11.1	11.1	10.9	
Other	6.2	6.1	6.4	6.7	6.9	7.2	7.4	7.6	7.8	7.8	8.1	8.6	9.1	
**Sex**														
Male	43.2	43.7	44.1	44.5	44.6	45.0	45.3	45.6	45.9	46.4	46.8	47.0	47.2	< 0.0001
Female	56.8	56.4	55.9	55.6	55.4	55.1	54.7	54.5	54.1	53.6	53.2	53.0	52.8	
**Region**														
Northeast	20.4	20.4	20.4	20.0	19.7	19.8	19.5	19.4	19.3	19.5	19.3	19.1	18.6	< 0.0001
Midwest	25.6	25.5	25.1	24.5	24.3	24.1	23.5	23.3	23.2	22.7	22.2	22.2	22.2	
South	39.9	40.0	39.9	40.6	40.8	40.7	41.3	41.4	41.4	41.8	42.2	42.1	42.1	
West	14.1	14.1	14.6	15.0	15.2	15.4	15.7	15.9	16.1	16.0	16.4	16.6	17.1	
**Community type**														
Large Central Metro	22.9	22.6	22.9	22.8	22.8	22.8	22.7	22.6	22.5	21.8	21.4	21.5	21.4	0.8562
Large Fringe Metro	22.0	22.3	22.7	22.9	23.0	23.2	23.3	23.4	23.6	23.9	24.1	24.1	24.0	
Medium Metro	21.8	21.7	21.5	21.4	21.4	21.2	21.3	21.3	21.3	21.4	21.5	21.4	21.5	
Small Metro	11.3	11.4	11.2	11.2	11.2	11.2	11.3	11.3	11.3	11.5	11.6	11.6	11.5	
Micropolitan	12.2	12.2	12.0	11.9	12.0	11.9	11.9	11.9	11.8	11.9	12.0	12.0	12.1	
Non-Core	9.8	9.8	9.8	9.8	9.7	9.6	9.7	9.6	9.5	9.5	9.5	9.5	9.5	
**Medicare/Medicaid dual eligibility**														
No	82.0	82.6	82.7	82.5	82.4	82.3	82.1	82.1	82.5	83.6	84.0	84.1	84.4	< 0.0001
Yes	18.0	17.4	17.3	17.5	17.6	17.8	17.9	17.9	17.5	16.5	16.0	15.9	15.7	

*Mantel-Haenszel Chi-square statistics were used, leveraging the ordinal nature of the year, age group, and community type variables

### Trends in prevalence of hypertension by community types across regions

**Fig. 1:**
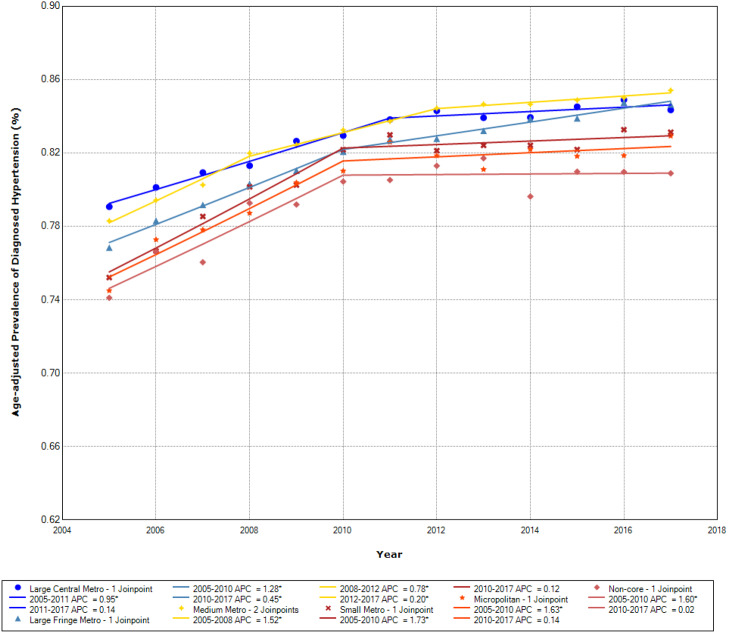
Age-adjusted prevalence of diagnosed hypertension among those with diabetes by community types in the Northeast

**Fig. 2:**
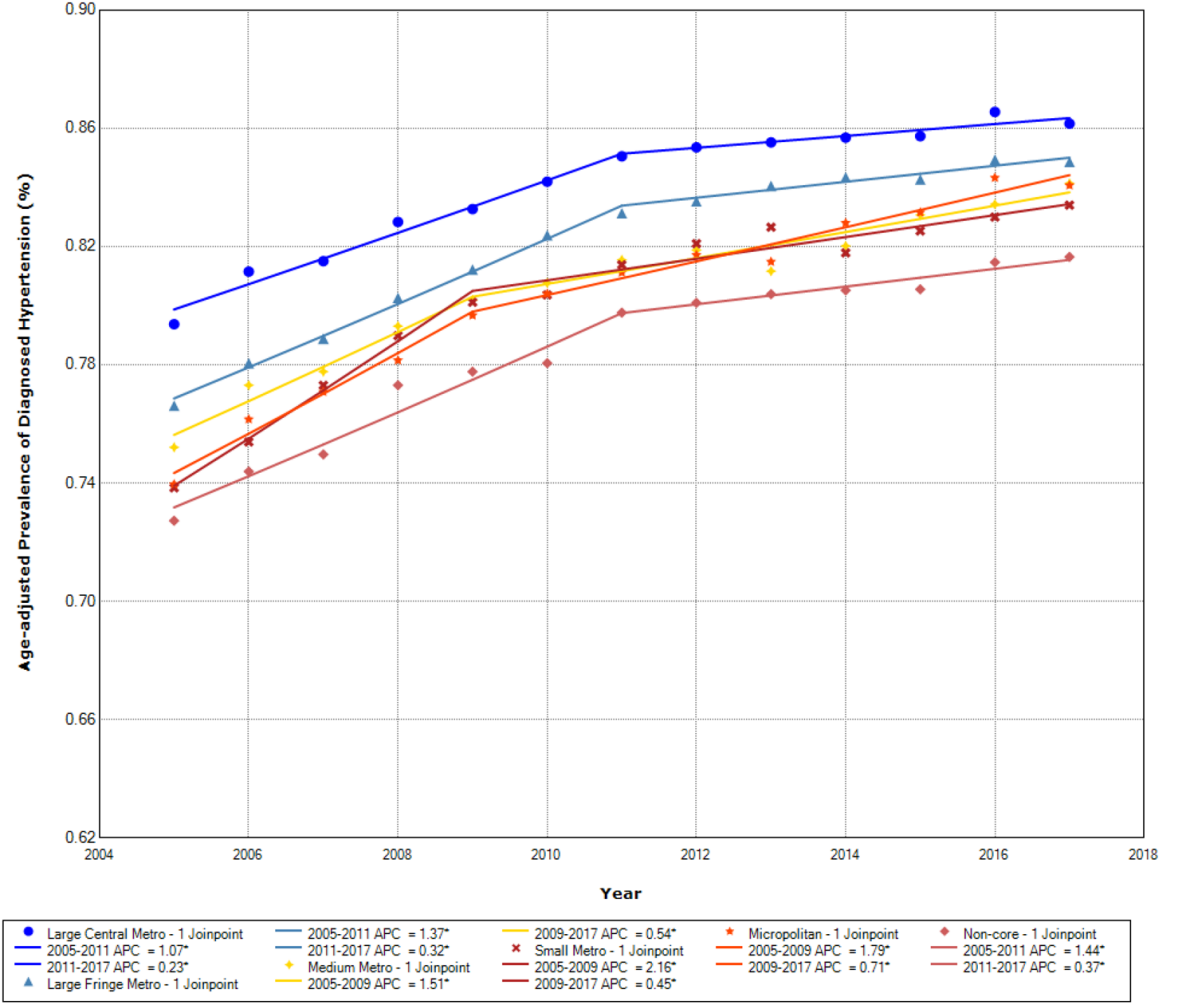
Age-adjusted prevalence of diagnosed hypertension among those with diabetes by community types in the Midwest

**Fig. 3:**
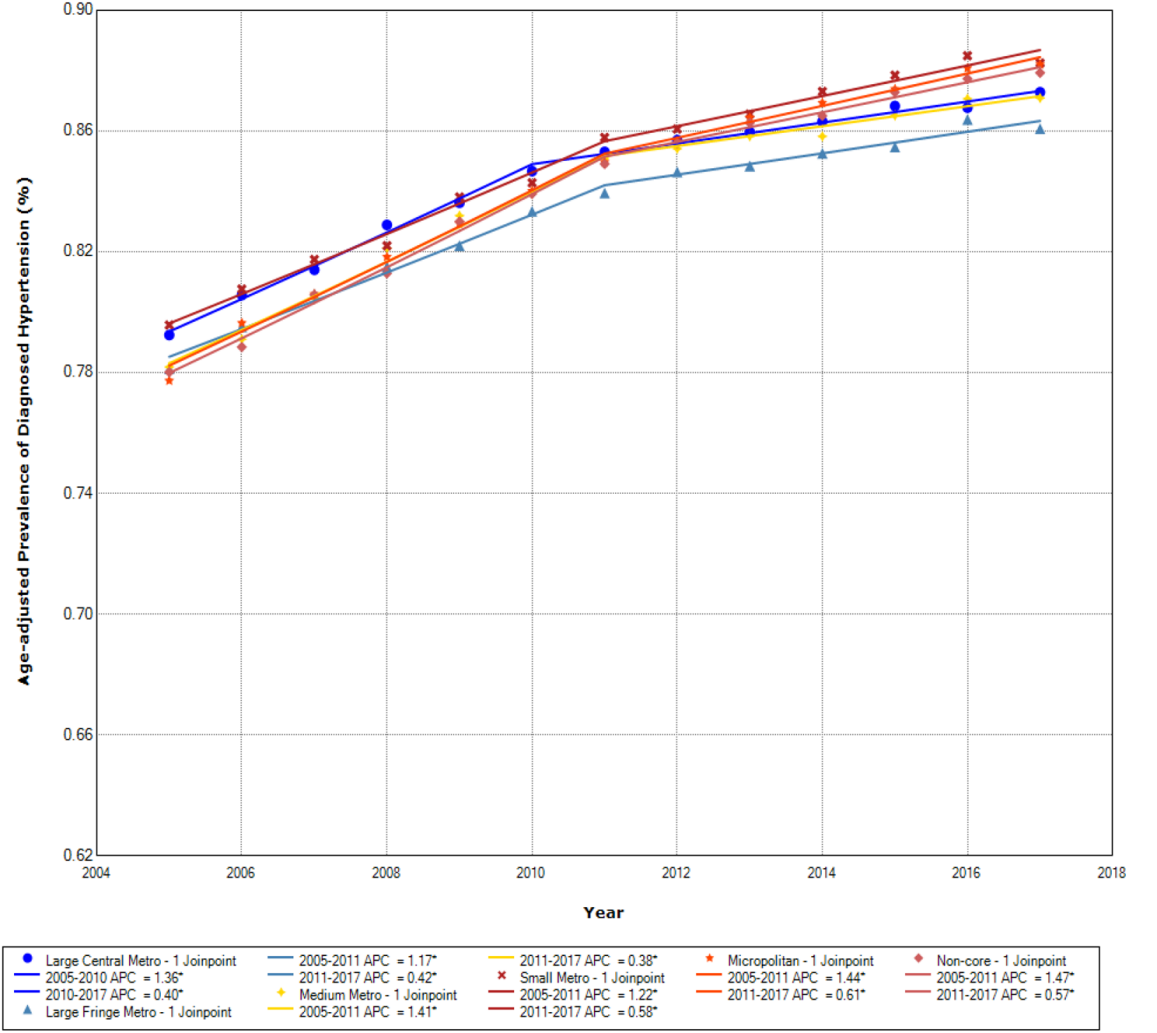
Age-adjusted prevalence of diagnosed hypertension among those with diabetes by community types in the South

**Fig. 4:**
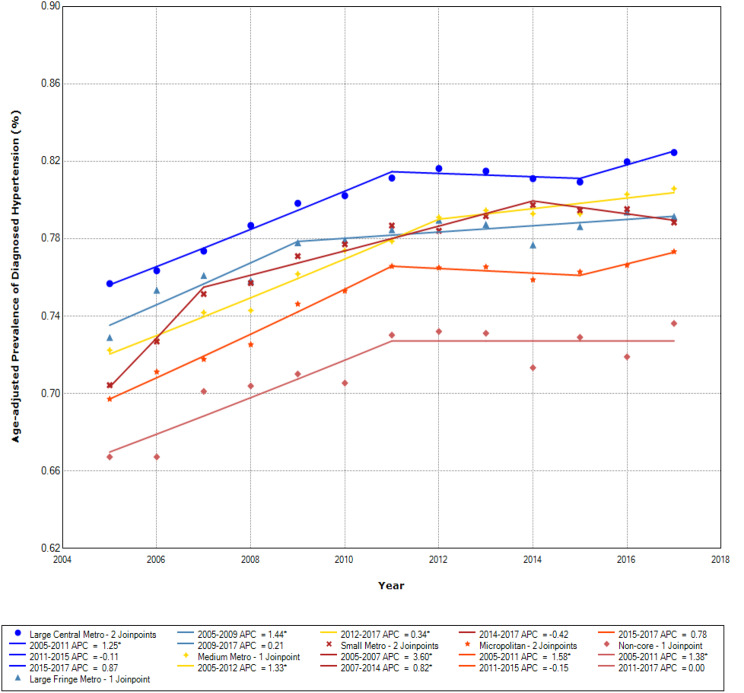
Age-adjusted prevalence of diagnosed hypertension among those with diabetes by community types in the West

The age-adjusted hypertension prevalence by rural-urban community types is presented for the Northeast, Midwest, South, and West regions, respectively, in Figs. 1, 2, 3 and 4. The prevalence of diagnosed hypertension among older adults with diabetes increased in all regions, although it differed across rural-urban community types.

In the Northeast region (Fig. 1), rural communities (non-core) had a lower prevalence of hypertension than more urban communities. From 2005 to 2010, the APC in hypertension prevalence was 1.28% in large fringe metropolitan areas, 1.73% in small metropolitan areas, 1.63% in micropolitan areas, and 1.60% in non-core areas. During 2010–2017, APCs were lower, ranging from 0.02% in non-core to 0.45% in large fringe metropolitan areas.

The prevalence of hypertension was higher in large central and large fringe metropolitan areas than those living in non-core areas in the Midwest region (Fig. 2). Between 2005 and 2011, the APC in hypertension was 1.07% in large central metropolitan areas, 1.37% in large fringe metropolitan, and 1.44% in non-core areas. The APC was lower in later years across all community types. Between 2011 and 2017, the APC in hypertension prevalence was 0.23% in large central metropolitan areas, 0.32% in large fringe metropolitan areas, and 0.37% in non-core areas.

Hypertension prevalence was generally higher in the South than in other regions. Additionally, differences in hypertension prevalence across rural-urban communities had smaller magnitudes in the South (Fig. 3). Overall, from 2005 to 2017, the APC in hypertension prevalence was higher in earlier years and there was a slowdown in later years across all rural-urban community types. For example, during 2005–2011, the APC was 1.17% in large fringe metropolitan areas, 1.41% in medium metropolitan areas, and 1.47% in non-core areas. During 2011–2017, the APC was 0.42% in large fringe metropolitan areas, 0.38% in medium metropolitan areas, and 0.57% in non-core areas. Interestingly, the rural-urban prevalence gradient in the South region essentially flips through the study period. In general, the prevalences in the beginning were higher in urban counties than in rural counties, but by the end of the study period, prevalences were higher in rural counties compared to urban.

In the West, hypertension prevalence was generally lower than other regions (Fig. 4). The prevalence of hypertension was highest in large central metropolitan areas and lowest in non-core areas. During 2005–2011, the APC was 1.25% in large central metropolitan areas and 1.38% in non-core areas. In later years, the APC was lower (APC = 0.00 in non-core areas in 2011–2017 and APC = 0.21 in large fringe metropolitan in 2009–2017).

### Association of rural-urban community type with hypertension prevalence

Prevalence ratios (PR) and 95% confidence intervals estimating hypertension prevalence across community types and by geographic region are presented in Table 2. In the unadjusted model (Model 1), compared to participants residing in large central metropolitan areas, older adults from non-core areas had a lower hypertension prevalence (Northeast PR = 0.958; Midwest PR = 0.934; South PR = 0.993; and West PR = 0.893). However, after adjustment for potential covariates including age, sex, race, Medicare-Medicaid dual-eligibility, and social vulnerability index, associations were weakened to either non-significant or close to 1, indicating very small community type differences in the prevalence of diagnosed hypertension (for example, in non-core areas, Northeast PR = 0.994; Midwest PR = 0.988; South PR = 1.001; and West PR = 0.935), though still statistically significant in the Midwest and West.

**Table 2:** Prevalence ratios of diagnosed hypertension for beneficiaries with diabetes living in different community types (versus Large Central Metro), Medicare 2005–2017

		Model 1 Urban-Rural Only		Model 2 Model 1 + Covariates		Model 3 Model 2 + SVI	
		PR (95% CI)	P	PR (95% CI)	P	PR (95% CI)	P
**Northeast**							
**Urban-Rural Classification**	Large Fringe Metro	0.993 (0.989–0.998)	0.0025	1.013 (1.009–1.018)	< 0.0001	1.025 (1.019–1.031)	< 0.0001
	Medium Metro	1.003 (0.998–1.009)	0.2284	1.024 (1.019–1.030)	< 0.0001	1.032 (1.026–1.039)	< 0.0001
	Small Metro	0.977 (0.970–0.985)	< 0.0001	1.001 (0.993–1.009)	0.8168	1.011 (1.002–1.020)	0.0162
	Micropolitan	0.970 (0.962–0.978)	< 0.0001	0.996 (0.987–1.004)	0.3054	1.006 (0.997–1.015)	0.2286
	Non-Core	0.958 (0.948–0.969)	< 0.0001	0.983 (0.973–0.994)	0.0025	0.994 (0.983–1.006)	0.3298
	Overall^*^		< 0.0001		< 0.0001		< 0.0001
**Midwest**							
**Urban-Rural Classification**	Large Fringe Metro	0.979 (0.975–0.984)	< 0.0001	1.003 (0.998–1.008)	0.2440	1.035 (1.029–1.041)	< 0.0001
	Medium Metro	0.961 (0.956–0.967)	< 0.0001	0.982 (0.977–0.986)	< 0.0001	1.003 (0.997–1.009)	0.3309
	Small Metro	0.955 (0.950–0.961)	< 0.0001	0.980 (0.974–0.986)	< 0.0001	0.999 (0.993–1.005)	0.7430
	Micropolitan	0.955 (0.950–0.960)	< 0.0001	0.983 (0.978–0.989)	< 0.0001	1.002 (0.996–1.009)	0.4415
	Non-Core	0.934 (0.928–0.929)	< 0.0001	0.962 (0.956–0.968)	< 0.0001	0.988 (0.981–0.995)	0.0006
	Overall^*^		< 0.0001		< 0.0001		< 0.0001
**South**							
**Urban-Rural Classification**	Large Fringe Metro	0.988 (0.984–0.991)	< 0.0001	0.997 (0.994–1.001)	0.1105	1.004 (1.000-1.008)	0.0323
	Medium Metro	0.992 (0.989–0.996)	< 0.0001	1.002 (0.998–1.005)	0.3412	1.003 (0.999–1.007)	0.0762
	Small Metro	1.006 (1.001–1.010)	0.0084	1.016 (1.012–1.020)	< 0.0001	1.017 (1.013–1.021)	< 0.0001
	Micropolitan	0.997 (0.993–1.001)	0.1725	1.006 (1.002–1.010)	0.0062	1.004 (0.999–1.008)	0.0638
	Non-Core	0.993 (0.989–0.997)	0.0012	1.002 (0.997–1.006)	0.4881	1.001 (0.997–1.005)	0.7109
	Overall^*^		< 0.0001		< 0.0001		< 0.0001
**West**							
**Urban-Rural Classification**	Large Fringe Metro	0.971 (0.964–0.979)	< 0.0001	0.986 (0.978–0.993)	0.0002	1.005 (0.997–1.013)	0.2355
	Medium Metro	0.967 (0.962–0.973)	< 0.0001	0.982 (0.976–0.988)	< 0.0001	0.985 (0.979–0.990)	< 0.0001
	Small Metro	0.962 (0.955–0.970)	< 0.0001	0.984 (0.977–0.992)	< 0.0001	0.985 (0.978–0.993)	0.0001
	Micropolitan	0.934 (0.926–0.942)	< 0.0001	0.958 (0.950–0.967)	< 0.0001	0.963 (0.955–0.971)	< 0.0001
	Non-Core	0.893 (0.882–0.904)	< 0.0001	0.916 (0.904–0.927)	< 0.0001	0.935 (0.923–0.946)	< 0.0001
	Overall^*^		< 0.0001		< 0.0001		< 0.0001

PR = Prevalence Ratio, 95% CI = 95% Confidence Interval

^*^Overall *P*-value indicates the 5 df joint likelihood ratio test of any difference between the NCHS urban-rural classification groups

*Note* Model 1 = NCHS Urban-Rural Classification; Model 2 = Model 1 + age, sex, race, and Medicare-Medicaid dual eligibility; Model 3 = Model 2 + social vulnerability index (SVI)

## Discussion

This study identified regional and community-level trends in diagnosed hypertension among older adults with diabetes during 2005–2017. Overall, hypertension prevalence increased in each region during the study period, with larger increases occurring from 2005 to around 2010. Additionally, differences in hypertension prevalence by rural-urban community type were observed, although these differences were smaller in the South region than other regions. The prevalence of hypertension was lower in non-core areas than large central metropolitan areas in each region, although these associations were attenuated after multivariable adjustment and the effect estimates were small.

The joinpoint regression analysis revealed interesting changes in the slope of the prevalence trends of hypertension over time for urban/rural categories across all geographic regions. We noticed a larger increase in hypertension prevalence occurring from 2005 to around 2010/2011 and a slowdown in the increase in more recent years (e.g., 2011–2017). One potential reason for this change in the prevalence trend could be healthcare reform in 2011, which has a spillover effect on the health outcomes of Medicare beneficiaries. Since the implementation of the Affordable Care Act (ACA) in 2011, some studies have shown increased utilization of certain clinical preventive services, including blood pressure monitoring among Medicare beneficiaries [26, 27]. This is partly because the ACA required most insurance plans and Medicare to cover a range of clinical preventive services without cost-sharing. The inncreased access to preventive services owing to ACA spillover effect may result in better monitoring of high blood pressure and a slowdown in the increase in hypertension prevalence among Medicare beneficiaries in the post-ACA period.

Our analysis found small differences in hypertension prevalence by rural-urban areas. We observed that prevalence estimates were relatively smaller in rural and larger in large metropolitan areas. In contrast, prior studies have reported mixed results, with most studies showing an excess burden of hypertension in rural areas and a few studies showing higher prevalence of hypertension in medium-lower metropolitan areas. For instance, using the similar urban-rural classification that we used, a study using data from the Behavioral Risk Factor Surveillance System (BRFSS) has shown that the age-standardized prevalence of self-reported hypertension was higher in the most rural counties (e.g., non-core and micropolitan) compared to large central metropolitan areas [28]. In a cohort study of adults 45 years or older, those residing in rural areas had a higher prevalence of hypertension than those residing in large central metropolitan areas [7]. Further, using a condensed NCHS rural-urban classification scheme, a recent study based on data from the 2013–2018 National Health and Nutrition Examination Survey (NHANES) reports that hypertension (defined as blood pressure (BP) ≥ 140/90) prevalence was higher for adults residing in medium to small metropolitan areas but not for those residing in most rural areas [9]. In contrast, our study found that among older adults with diabetes, hypertension prevalence was generally lower for those in rural areas than in urban areas within each geographic region, except for the South.

Overall, our analysis stratified by region shows a higher prevalence of age-adjusted hypertension in urban than rural areas in all regions except for the South, where hypertension prevalence is largely similar across community types. Such findings of rural-urban disparities in hypertension by region are consistent with a recent study that examined trends in hypertension-related cardiovascular mortality rates from 2007 to 2017. While hypertension-related mortality rates increased in rural and urban areas during the study period, the hypertension-related mortality rate was higher among those in rural areas in the South [16]. Our analysis did not specifically examine mortality-related outcomes, but we note that a higher prevalence of hypertension in the South is consistent with previous studies [10, 29].

Contrary to some previous studies, our analysis found that rural (i.e., non-core) areas generally had lower hypertension prevalence than urban (i.e., large central metropolitan) areas. To better contextualize our observed higher estimates in urban areas, we note a few methodological and measurement differences in self-report survey data and insurance claims that differ in estimating hypertension. Firstly, validation studies generally report that estimates of hypertension tend to be significantly higher in self-reported surveys than in insurance claims data [30]. Accuracy of self-reports of chronic conditions may often be limited due to social or personal stigma associated with the conditions and by the presence of other debilitating conditions such as cognitive impairment or severe mental health problems [31]. Secondly, another potential reason we observed a different association than existing studies is that our study population is Medicare beneficiaries with diabetes. In contrast, most previous studies looked at hypertension among the general population without any disease condition. Future studies can examine whether hypertension prevalence differs between older adults with and without diabetes, especially across urban-rural classification.

Thirdly, unlike most previous studies, we chose to stratify our analysis by region to account for the potential confounding by region [10, 32]. Due to confounding by region, we found that urban areas had a higher prevalence in most regions. Finally, administrative claims data is also not without limitations; an insurance claim does not necessarily represent a disease status. At best, an insurance claim represents an episode of care for which a claim was submitted. Most importantly, as access to healthcare facilities is better in urban than rural areas, episodes of care are likely to be higher in urban areas. We note that in light of these methodological and measurement differences, our study findings should be interpreted and compared to existing studies.

Understanding the potential reasons for regional differences in the burden of hypertension is important for the effective prevention and management of chronic conditions. We note that the impact of broader contextual factors, such as community-based economic inequality, lifestyle, healthcare, and environmental factors, may differ across regions and thus differentially affect the burden of chronic disease [32–34]. Because our findings demonstrate disparities in the prevalence of hypertension in regional and urban-rural settings, future studies might investigate how underlying community characteristics may shape differences in chronic disease conditions within these contexts.

Our study has a few limitations. First, our analysis is limited to Medicare fee-for-service beneficiaries and does not represent those in Medicare Advantage or other commercial insurance plans. The financial incentives and reimbursement structures vary between Medicare fee-for-service and Medicare Advantage. These differences may affect the rate of diagnosis of hypertension. Second, this analysis is based on Medicare administrative claims data that reflect a disease diagnosis and not the prevalence of the condition itself, as those with undiagnosed hypertension or undiagnosed diabetes would not be captured using claims data. Third, the rural-urban classification used was at the county level. Because defining rurality is a complex phenomenon, its classification should represent a nuanced matrix of geographical and population-level characteristics [35]. Some counties may have urbanized and non-urbanized pockets because of their size, population density, and other area-level characteristics; thus, obtaining and characterizing such a granular rural-urban continuum within a county boundary is challenging. Finally, we could not examine the prevalence of hypertension stratified by race/ethnicity in rural-urban communities due to the smaller sample size in some race/ethnicity categories.

In summary, among older adults with diabetes, the prevalence of diagnosed hypertension in most regions was higher for Medicare beneficiaries residing in urban areas than rural areas. The differential prevalence of diagnosed hypertension in rural-urban communities across geographic regions has implications for targeted interventions to improve chronic disease prevention and management. Although we found smaller yet statistically significant differences, further investigation is needed to examine why older adults living in certain community types have a higher burden of chronic diseases. Understanding underlying contextual and service delivery factors may be immensely useful for designing community-specific preventive interventions to reduce place-based disparities in chronic diseases.

### Electronic supplementary material

Below is the link to the electronic supplementary material.

Supplementary Material 1

## Data Availability

The data that support the findings of this study are available from the Data Assistance Center (ResDAC) (https://resdac.org), but restrictions apply to the availability of these data, which were used under license for the current study, and so are not publicly available.
