# Supplementary material for: Regional and rural-urban patterns in the prevalence of diagnosed hypertension among older U.S. adults with diabetes, 2005–2017
Source: BMC Public Health. 2024 May 16;24:1326. doi: 10.1186/s12889-024-18802-5 (PMC11100106; doi:10.1186/s12889-024-18802-5)
Supplement: Supplementary file 1 — Supplementary Material 1 [file 12889_2024_18802_MOESM1_ESM.docx]

**Supplementary Table S1: Diagnosis codes used to define diabetes and hypertension in the Medicare data**

| **Diabetes** | |
| --- | --- |
| ICD-9* | 250.XX**, 357.2, 362.0X, 366.41 |
| ICD-10* | E0836, E0842, E0936, E0942, E1010, E1011, E1029, E10311, E10319, E1036, E1039, E1040, E1042, E1051,  E10618, E10620, E10621, E10622, E10628, E10630, E10638, E10641, E10649, E1065, E1069, E108, E109, E1100,  E1101, E1129, E11311, E11319, E11329, E11339, E11349, E11359, E1136, E1139, E1140, E1142, E1151, E11618,  E11620, E11621, E11622, E11628, E11630, E11638, E11641, E11649, E1165, E1169, E118, E119, E1310, E1336,  E1342, E1037X1, E1037X2, E1037X3, E1037X9, E1110, E1111, E113291, E113292, E113293, E113299, E113391,  E113392, E113393, E113399, E113491, E113492, E113493, E113499, E113591, E113592, E113593, E113599,  E1137X2 |
| **Hypertension** | |
| ICD-9 | 401.X, 403.0X, 403.1X, 403.9X |
| ICD-10 | I10, I120, I129, I16.X |

Number of Claims Requirement

For Medicare carrier provider and inpatient hospital data, at least one claim with diagnosis as listed in the above code list. For outpatient hospital data, at least two claims that were at least 7 days apart with diagnosis as listed in the above code list.

**ICD-9 before October 1, 2015 and ICD-10 October 1, 2015 and after*
